# Supplementary material for: Complexome profiling of the Chlamydomonas psb28 mutant reveals TEF5 as an early PSII assembly factor
Source: Plant Cell. 2025 Mar 17;37(6):koaf055. doi: 10.1093/plcell/koaf055 (PMC12164590; doi:10.1093/plcell/koaf055)
Supplement: koaf055_Supplementary_Data [file koaf055_supplementary_data.zip › psb28_interactive.zip!/plots/intensity/qualityPlots/peptides/CPLD10_Cre13.g578650.t1.1.html]

### Cre13.g578650.t1.1

Trivial name: CPLD10  
SeqCoverage [median]: 3.80  
Mass: 66.14 kDa  
  
 

| Sequence | MissedCleavage | Mass [Da] | wtIntensity | muIntensity | Score |
| --- | --- | --- | --- | --- | --- |
| TAGTEGLVLR | 0 | 1015.57 | 508.59 | 618.55 | 122.13 |
|  |  |  | 508.6 | 618.5 |  |
